# Prescription Pain Reliever Use and Misuse Among Marijuana Users Age 50+ Years

**DOI:** 10.1093/geroni/igaa057.178

**Published:** 2020-12-16

**Authors:** Namkee Choi, Diana DiNitto

**Affiliations:** 1 University of Texas, Austin, Texas, United States; 2 University of Texas at Austin, Austin, Texas, United States

## Abstract

Marijuana use among individuals aged 50+ has steadily increased over the past decade, with 8% reporting past-year use in 2018. National epidemiologic data also showed a 36% rate of past-year prescription pain reliever (PPNR) use in the 50+ age group in 2018, a decrease from 40% in 2015, but still significantly higher than for younger age groups. Little research has, however, focused on older adults’ dual recreational and/or medical marijuana and PPNR use. This study, based on the 2015-2018 National Survey of Drug Use and Health, examined rates and correlates of dual marijuana and PPNR use and misuse among those aged 50+ who reported past-year marijuana use (N=2,632). Our findings showed that 43.6% of past-year marijuana users did not use any PPNR, 47.1% used PPNR but did not misuse, and 9.4% misused PPNR in the past year, showing that one in six dual marijuana and PPNR users reported misusing PPNR. The risks of PPNR use/no misuse and PPNR misuse were higher among those who had more chronic medical conditions and major depressive episode. Additionally, the risk of PPNR use/no misuse was associated with high frequency and medical marijuana use; and the risk of PPNR misuse was associated with younger marijuana initiation age and marijuana and other illicit drug use disorders. Thus, correlates of dual marijuana and PPNR use/misuse among older adults are poor physical and mental health problems and problematic marijuana use. Older adults with marijuana and PPNR misuse need access to evidence-based treatments for pain management and substance misuse.

